# A Rat Model of Prenatal Zika Virus Infection and Associated Long-Term Outcomes

**DOI:** 10.3390/v13112298

**Published:** 2021-11-18

**Authors:** Morgan L. Sherer, Elise A. Lemanski, Rita T. Patel, Shannon R. Wheeler, Mark S. Parcells, Jaclyn M. Schwarz

**Affiliations:** 1 Department of Psychological and Brain Sciences, University of Delaware, Newark, DE 19716, USA; rdesai@udel.edu (R.T.P.); shwheeler98@gmail.com (S.R.W.); jschwarz@udel.edu (J.M.S.); 2 Department of Animal and Food Sciences, University of Delaware, Newark, DE 19716, USA; parcells@udel.edu

**Keywords:** Zika virus (ZIKV), neurodevelopment, congenital infection, neurogenesis, hippocampus, interferon

## Abstract

Zika virus (ZIKV) is a mosquito-borne flavivirus that became widely recognized due to the epidemic in Brazil in 2015. Since then, there has been nearly a 20-fold increase in the incidence of microcephaly and birth defects seen among women giving birth in Brazil, leading the Centers for Disease Control and Prevention (CDC) to officially declare a causal link between prenatal ZIKV infection and the serious brain abnormalities seen in affected infants. Here, we used a unique rat model of prenatal ZIKV infection to study three possible long-term outcomes of congenital ZIKV infection: (1) behavior, (2) cell proliferation, survival, and differentiation in the brain, and (3) immune responses later in life. Adult offspring that were prenatally infected with ZIKV exhibited motor deficits in a sex-specific manner, and failed to mount a normal interferon response to a viral immune challenge later in life. Despite undetectable levels of ZIKV in the brain and serum in these offspring at P2, P24, or P60, these results suggest that prenatal exposure to ZIKV results in lasting consequences that could significantly impact the health of the offspring. To help individuals already exposed to ZIKV, as well as be prepared for future outbreaks, we need to understand the full spectrum of neurological and immunological consequences that could arise following prenatal ZIKV infection.

## 1. Introduction

Zika virus (ZIKV) is a positive-stranded RNA virus within the Flaviviridae family and belongs to the same genus, *Flavivirus*, as Dengue, West Nile, and Yellow Fever viruses [1]. ZIKV was first discovered in 1947 from a sentinel rhesus macaque in Uganda and the virus remained in relative obscurity for decades until 2007, when it caused its first noteworthy epidemic on Yap island in Micronesia, and then in 2013 when it reached New Caledonia, French Polynesia, and most extensively, Brazil in 2015 [2,3]. The virus is primarily transmitted through an infection cycle between humans and the *Aedes aegypti* mosquito which inhabit tropical and sub-tropical regions such as Asia, Africa, and the Americas. ZIKV has also been shown to be transmitted through other routes including sexual transmission, transfusion of blood products, breast milk feeding, as well as vertical transmission from mother to fetus [4]. Only 20% of ZIKV infection results in the presentation of symptoms; and when symptoms do occur, they are typically mild and resolve in less than two weeks [1,5]. Clinical symptoms of ZIKV infection in adults are similar to other flavivirus infections (such as dengue fever) and include fever, headache, joint pain, muscle pain, and maculopapular rash. More severe clinical outcomes of ZIKV infection have been reported such as the neurological disorder Guillain–Barré syndrome; however, these consequences are comparatively very rare [1].

In contrast, congenital ZIKV infection results in more serious clinical outcomes. In October 2015, there was nearly a 20-fold increase in the incidence of microcephaly and birth defects seen among women giving birth in Brazil [6,7,8], leading the Centers for Disease Control and Prevention (CDC) to officially declare a causal link between prenatal ZIKV infection and the serious brain abnormalities seen in affected infants [9]. At that time, Congenital Zika Syndrome (CZS) was defined as an in utero ZIKV infection associated with severe microcephaly in which the skull has partially collapsed, decreased brain tissue with a specific pattern of brain damage (including subcortical calcifications), damage to the back of the eye (including macular scarring and focal pigmentary retinal mottling), congenital contractures (clubfoot or arthrogryposis), hypertonia, or restricted body movement soon after birth [10]. This definition is controversial and is still evolving, for example, many subsequent case reports have demonstrated that microcephaly is not consistently present in CZS [5]. Further, it has been hypothesized that microcephaly may be just the “tip of the iceberg” for neurological and cognitive consequences associated with this virus. In fact, pediatricians are now reporting an increased risk of seizures, irritability, and cognitive developmental delays in ZIKV-affected children, some of whom appeared asymptomatic at birth [11,12]. It is therefore necessary that animal models are created that best mirror the symptoms, the transmission, and outcomes associated with this virus in order to understand the long-term neurological consequences of prenatal ZIKV infection in the affected offspring so that we may one day learn how to prevent or reverse these outcomes.

Since its emergence, there has been a significant increase in research dedicated to understanding the pathogenicity of ZIKV in order to develop vaccines and therapeutic strategies able to combat infection [13]. Nevertheless, no effective therapies exist. This is partially due to limitations in current ZIKV animal models used for experimentation, which do not accurately mimic human ZIKV infectivity. The most widely used animal models of prenatal ZIKV infection utilize immunocompromised mice such as interferon regulatory factor knockout mice and interferon receptor knockout or deficient mice [14,15,16]. These animal models frequently also use atypical routes of infection such as: direct intracranial administration of the virus, footpad injection, intraperitoneal injection, and intravenous administration [15,17,18]. These models are widely used because they allow for infection as opposed to adult wild-type (WT) C57BL/6, Swiss Webster, BALB/c, and the CD-1 mice strains, which are all unable to sustain ZIKV infection following typical peripheral inoculation with ZIKV [14,19,20]. Data from our lab demonstrated that rats are naturally immunocompromised during pregnancy, a phenomenon that is also observed in humans, mice, and other species [21,22]. As a result, we hypothesized that pregnant rats might be naturally vulnerable to maternal ZIKV infection, thus making them an ideal animal model for prenatal ZIKV infection. Therefore, we have developed a rat model to study the effects of ZIKV infection during pregnancy in order to examine the neurological outcomes on the offspring. In this rat model, pregnant females are inoculated on embryonic day 18 (E18), during late gestation when immunosuppression is greatest, using a subcutaneous injection with ZIKV.

It is well known that disruptions of early-life programming can permanently alter later-life neural function [23]. Mouse and non-human primate models have demonstrated neurological impairments associated with congenital ZIKV infection, including impairment in memory [24,25], vision [25], social behavior [25,26], emotional stress response [27], and motor coordination [25,26]. Other pathogens which are able to access the fetal compartment, such as Cytomegalovirus and *Toxoplasma gondii* have been shown to disrupt neurogenesis in the developing brain [28,29]. Emerging evidence suggests that ZIKV preferentially infects neural progenitor cells (NPCs), the precursor cells to neurons and glia [2,6,8,9]. Precise timing of NPC proliferation, as well as proper differentiation, neuronal migration, and maturation is critical for normal brain development [30]. Dysregulation of these cells has been shown to lead to severe impairments in cognition including spatial recognition, learning and memory, emotional regulation, and later onset mental health disorders such as schizophrenia, depression, and Alzheimer’s disease [31]. The dentate gyrus is one of the two restricted niches that contain NPCs and undergoes neurogenesis throughout the lifespan [32,33]. These niches are characterized by a high vascular density and proximity to cerebrospinal fluid (CSF), allowing for efficient communication with both signaling molecules and circulating viruses [34].

These data, along with previous findings from our lab which demonstrate reduced pup mortality, increased apoptosis, and cerebrocortical dysplasia in rat pups prenatally exposed to ZIKV [35] have led us to hypothesize that prenatal ZIKV exposure may permanently alter cellular proliferation, survival, and decrease life-long neurogenesis in the dentate gyrus of the hippocampus. We hypothesized that this could be caused by either persistent ZIKV residence in the brain, or alternatively by ZIKV-induced decreases in the NPC population early in life. Using our rat model of prenatal ZIKV infection, we differentiated between these possibilities by first determining the duration and location of ZIKV in the brain following prenatal infection, and then assessed cellular proliferation, survival, and neurogenesis in the adolescent brain. Understanding the impact of prenatal ZIKV exposure on these vulnerable hippocampal populations is crucial as resulting deficits may not manifest until higher-order processing emerges.

It is now recognized that ZIKV-related neurological complications can emerge even without the confirmed diagnosis of CZS, suggesting an unrecognized population of children who may have been impacted by prenatal exposure. However, the full spectrum of these long-term consequences remains unknown. Therefore, it is critical that we develop animal models that best replicate the human disease so that we may understand the long-term impact of prenatal ZIKV infection and may one day learn how to prevent or reverse them. The ‘Barker Hypothesis’ posits that the early-life environment determines the framework for later adult functioning [36]. Thus, we used our rat model of prenatal ZIKV infection to investigate whether ZIKV exposure during gestation leads to behavioral and molecular alterations later in life. The unique goals of the experiments presented here were to (1) determine the long-term location and duration of ZIKV in the rat brain of the affected offspring following prenatal infection, (2) determine whether prenatal ZIKV infection results in long-term deficits in cellular proliferation, survival, and neurogenesis in the juvenile rat brain, and (3) determine whether prenatal ZIKV infection results in long-term alterations in the offspring’s behavior and immune system. The data obtained from these experiments will allow scientists and clinicians to better understand the potential negative effects of prenatal ZIKV infection.

## 2. Materials and Methods

### 2.1. Experimental Subjects

Adult male and female Sprague Dawley rats were ordered from Envigo Laboratories in Indianapolis, Indiana. Rats were housed in same sex pairs in clear, polyethylene cages (45 cm × 20.5 cm × 24 cm) and allowed one week of acclimation to the facility prior to breeding. The colony room was maintained at 22 °C on a 12:12 h light:dark cycle (lights on at 7:00 a.m.) and all rats had ad libitum access to food and water. For breeding, male and female pairs were housed together for 24 h and the presence of sperm plugs was checked to determine the date of conception, designated as embryonic day one (E1). On E18, pregnant females were transported to a Biosafety Level 2 (BSL2) animal isolation facility where they were individually housed for the remainder of their pregnancy. Litters were culled to 4 males and 4 females in order to ensure similar access to maternal food and care among all pups. Pups were weaned into separate same-sex cages on postnatal day 21 (P21). One male and one female from each litter was used for each experiment: rotarod, BrdU analysis, and poly(I:C) assessment. Sentinel rats were housed in the colony room and periodically examined for the presence of common rodent diseases. All tests came back negative. All experiments were approved by the University of Delaware Institutional Animal Care and Use Committee (Animal Use Protocol #1306).

### 2.2. ZIKV Growth Conditions

Vero C1008 cells (ATCC^®^ CRL-1586^TM^) were grown in Dulbecco’s Minimum Essential Medium (DMEM), high glucose with 10% fetal bovine serum, 4 mM L-glutamine, 1% penicillin/streptomycin/neomycin (PSN), and 0.5% fungizone and incubated at 37 °C with 5% CO_2_. The human Zika virus (ZIKV) isolate Puerto Rico (December 2015) strain PRVABC59, ATCC^®^ VR-1843, GenBank Accession: KU501215) was used for all infection studies. Zika was propagated in T75 flasks of Vero cells at an MOI of ~1 in Vero cells and collected at 96 h post-infection. ZIKV stocks were collected via three freeze/thaw cycles (−80 °C to a 37 °C water bath), clarified via centrifugation at 3500 rpm for 10 min to remove cellular debris, and aliquots were frozen at −80 °C and then stored in liquid nitrogen. At the time of harvest, 25% of the viral stock (~5 mls) was UV inactivated in an open 100 mm dish in a Stratalinker UV-crosslinker at a setting of 200 (20,000 mJoules) for 10 min. ZIKV stocks and UV-inactivated stocks were titrated on Vero cells, fixed at 96 hrs post-infection and the TCID50 was determined by IFA. This work was carried out under IBC protocol #16-021. 

### 2.3. ZIKV Infection

Pregnant females were inoculated through subcutaneous injection on their dorsum on E18 with either a diluent control (0.1 mL of the same culture media used to grow ZIKV) or ZIKV (dose of 107 PFU in 0.1 mL culture media). Embryonic time point and ZIKV dose were selected based on work that was previously published in our lab [35]. Immediately following injections rats were returned to their home cage and left undisturbed until the start of additional experimental procedures.

### 2.4. Maternal Behavior

On P1, the day after birth, maternal behavior was assessed by recording naturally occurring mother-young behavioral interactions. The goal was to assess whether maternal ZIKV infection just 6 days prior results in significant deficits in maternal behavior that could adversely affect the offspring. The 30 min home-cage observation sessions took place once in the morning and once in the afternoon by observers who were blind to treatment groups. Behaviors were quantified and separated into “pup-directed behaviors” (i.e., licking, hovering, nursing, approaching, transporting, and retrieving) and “non-pup-directed behaviors” (i.e., exploring, eating, drinking, grooming, wall climbing, and tail chasing). The incidence of pup-directed and non-pup-directed behaviors was calculated for each dam. Average incidence was calculated for each group and was analyzed as a function of infection using a one-way ANOVA.

### 2.5. Rotarod

In the rotarod task [37], rats were acclimated to the testing room for 20 min prior to testing on P24/P60. Three trials per rat were conducted on each day. Rats were placed on the accelerating rotarod (MedAssociates, Fairfax, VT, USA) at a speed of 4 rotations per minute (rpm) that gradually increased from 4 to 40 rpm over a total of 5 min. Infrared beams were used to quantify the latency of a rat to fall off the rod. Rats were placed in their home cage for at least 2 min between each trial to rest. The latency to fall and the rod speed value were recorded. Statistical analysis was conducted using SPSS software. A two-way ANOVA was used for analysis, with infection and sex as factors. Following the last behavioral task (P60), rats were sacrificed, and brain and serum samples were collected and used for the detection and quantification of ZIKV.

### 2.6. BrdU Administration

One male and one female pup from each litter was injected with 5-bromo-2′-deoxyuridine (BrdU, 100 mg/kg) intraperitoneally once a day for three consecutive days as juveniles (P24–26) [38]. Half of the rats were sacrificed 24 h following the last injection (on P27) to examine the number of proliferating cells during this time. The remainder of the rats were sacrificed 2 weeks after the last BrdU injection (on P40) to examine the survival rate of the cells generated and labeled with BrdU during those three days as well as their differentiation into neurons during this time.

### 2.7. Immunohistochemistry

At 24 h or 2 weeks following the last BrdU injection, rats were euthanized by administration of an overdose of Euthasol (ANADA 200-071) via i.p. injection. Once anesthetized, rats were perfused via cardiac puncture with ice-cold, 0.9% saline solution to remove blood and peripheral immune cells from the brain, and then subsequently perfused with 4% ice-cold paraformaldehyde (PFA). Whole brains were collected on ice and immediately fixed in 4% PFA for 24 h at 4 °C. Whole brains were then transferred to fresh 4% PFA, then cryoprotected in 30% sucrose solution, and finally fresh 30% sucrose solution at 24 h intervals and stored at 4 °C.

Whole brains were sliced at 40µm on a Leica cryostat at −25 °C into wells containing 0.001% Sodium Azide Solution. Sliced brains were stored at 4 °C until staining. BrdU, a biomarker for proliferating cells, and NeuN, a biomarker for neurons, were chosen as the target proteins for staining. One out of every five sections in series were washed 3 times (at least 5 min each) with phosphate-buffered saline (PBS) and then underwent methanol quenching by incubating for 30 min at room temperature in 50% methanol. Sections were washed again in PBS (three times for at least 5 min each). Samples were then incubated for 25 min at 37 °C in 2 N hydrochloric acid. Samples were washed again in PBS (three times for at least 5 min each). Samples were then incubated in blocking solution for 1 h containing PBS, normal goat serum (Vector Laboratories, Burlingame, CA, USA), and 30% Triton X (Fisher Scientific, Waltham, MA, USA). Sections were then incubated with primary antibodies (rat anti-BrdU, 1:500; Accurate Chemical, Carle Place, NY, USA; mouse anti-NeuN, 1:500; Sigma, St. Louis, MO, USA) overnight at 4 °C.

On day 2, sections were washed (three times for at least 5 min each) and incubated with fluorescent secondary antibodies (Alexa Fluor^TM^ 568 goat anti-rat IgG, 1:200; Invitrogen; Alexa Fluor^TM^ 647 goat anti-mouse IgG, 1:200; Invitrogen) for 2 h at room temperature in the dark. Sections were washed (three times for at least 5 min each) and mounted on Superfrost++ Micro Slides (Fisher Scientific, Waltham, MA, USA), cover slipped (1.5; VWR, Radnor, PA, USA) with VECTASHIELD^®^ Antifade mounting medium with DAPI (Vector Laboratories, Burlingame, CA, USA) in the dark and stored at 4 °C until analysis.

### 2.8. Confocal Imaging and Imaris Analysis

Confocal fluorescent images were acquired on a Zeiss 880 confocal microscope equipped with 405, 458, 488, 514, 561, 633, and 680–1080 lasers using ZEN imaging software. This microscope is also equipped with an Airyscan detector which enables super-resolution and high-speed imaging. Confocal z stacks with 2 µm z intervals were tiled to include the dentate gyrus of the hippocampus using a 20× objective. Ten dentate gyrus sections were imaged per animal, with 5–7 animals in each group. Imaged z stacks were Airyscan processed, stitched together, and uploaded into Imaris (BitPlane) to create a three-dimensional rendering of the dentate gyrus. The dentate gyrus was selected using the surfaces module to create a volumetric boundary of the region. Using the ‘spots’ function in the Imaris software, positively labeled BrdU cells were counted and the number of those cells positively colocalized with labeled NeuN cells were measured. BrdU+ density was calculated as the total number of positively labeled BrdU+ cells/volume (µm^3^). BrdU+/NeuN+ colocalization density was calculated as number of colocalized cells/volume (µm^3^).

### 2.9. Poly(I:C) Injection

The 4th male and female per litter were used to test immune reactivity following a second immune challenge in adulthood. Poly(I:C) is a commonly used viral mimetic that is recognized by the pattern recognition receptor, Toll-like receptor (TLR) 3, which specifically recognizes double stranded RNA, the genetic information for many viruses [39]. On P60, one male and one female from each litter were given a low dose (1 mg/kg) [40,41,42] of the viral immunostimulant, polyinosinic:polycytidylic acid (poly(I:C)) sourced from Sigma. Animals were given poly(I:C) diluted in 0.9% sterile saline via intraperitoneal injection. Six hours later, the peak of the poly(I:C) response [43], rats were sacrificed and serum, spleen, and brain were collected for the analysis of peripheral and central cytokine/immune cell analysis.

### 2.10. Real-Time PCR

Three different time points were assessed for ZIKV presence: P2, P24, and P60 using brain and serum samples collected at the time of euthanasia. Brain samples and P2 serum samples were extracted using Isol-RNA lysis reagent, P24 and P60 serum samples were extracted using the QIAamp Viral RNA Minikit. Viral RNA was quantified by qRT-PCR using the primers and probe previously published [44] The RT-PCR was performed using the iTaq Universal OneStep RT-qPCR kit on an CFX96Touch real-time PCR machine. Viral presence was assessed by comparing samples to a standard curve generated using serial 10-fold dilutions of synthetic ZIKV RNA (ATCC).

Immune molecules related to the inflammatory response were measured using real-time PCR including *Il-1β*, *IL-6*, *IL-4*, *TNF-α*, *IFN-β*, *Iba1*, and *COX-2*. RNA was extracted from brain and spleen samples from rats using Isol-RNA lysis reagent and cDNA was synthesized from extracted RNA using the QuantiTect^®^ Reverse Transcription Kit. Relative gene expression was quantified by real-time PCR using the RealMasterMix^TM^ Fast SYBR Kit on a CFX96Touch real-time PCR machine (see Table 1 for primer sequences). HPRT1 was used as the housekeeping gene for all experimental groups as it does not differ significantly by sex, treatment, or time point. Samples were run in duplicate on real-time PCR plates. For each reaction, the average quantitative threshold amplification cycle number (Cq) value was determined from each duplicate, and the 2−ΔΔCq method was used to calculate the relative gene expression for each gene of interest relative to the housekeeping gene. A two-way ANOVA, with treatment and sex as factors, were run for each gene of interest.

### 2.11. Analysis of Gene Expression following Treatment with Poly(I:C)

Immune molecules related to the inflammatory response were measured using real-time PCR including *Il-1β*, *IL-6*, *IL-4*, *TNF-α*, *IFN-β*, *Iba1*, and *COX-2*. RNA was extracted from brain and spleen samples from rats using Isol-RNA lysis reagent and cDNA was synthesized from extracted RNA using the QuantiTect^®^ Reverse Transcription Kit. Relative gene expression was quantified by real-time PCR using the RealMasterMix^TM^ Fast SYBR Kit on a CFX96Touch real-time PCR machine (see Table 1 for primer sequences). HPRT1 was used as the housekeeping gene for all experimental groups as it does not differ significantly by sex, treatment, or time point. Samples were run in duplicate on real-time PCR plates. For each reaction, the average quantitative threshold amplification cycle number (Cq) value was determined from each duplicate, and the 2−ΔΔCq method was used to calculate the relative gene expression for each gene of interest relative to the housekeeping gene. A two-way ANOVA, with treatment and sex as factors, were run for each gene of interest.

## 3. Results

The current study explored long-term behavioral and molecular alterations following gestational ZIKV exposure in a rat model of prenatal ZIKV infection. We determined the long-term location and duration of ZIKV in the rat brain of the affected offspring using RT-qPCR, examined cellular proliferation survival, and neurogenesis in the juvenile rat brain using immunofluorescence, measured the effect of prenatal ZIKV infection on maternal and offspring behavior using maternal observation and the rotarod task. and examined the effect of poly(I:C) administration on the offspring immune response using RT-qPCR.

### 3.1. Maternal Behavior

**Figure 1:**
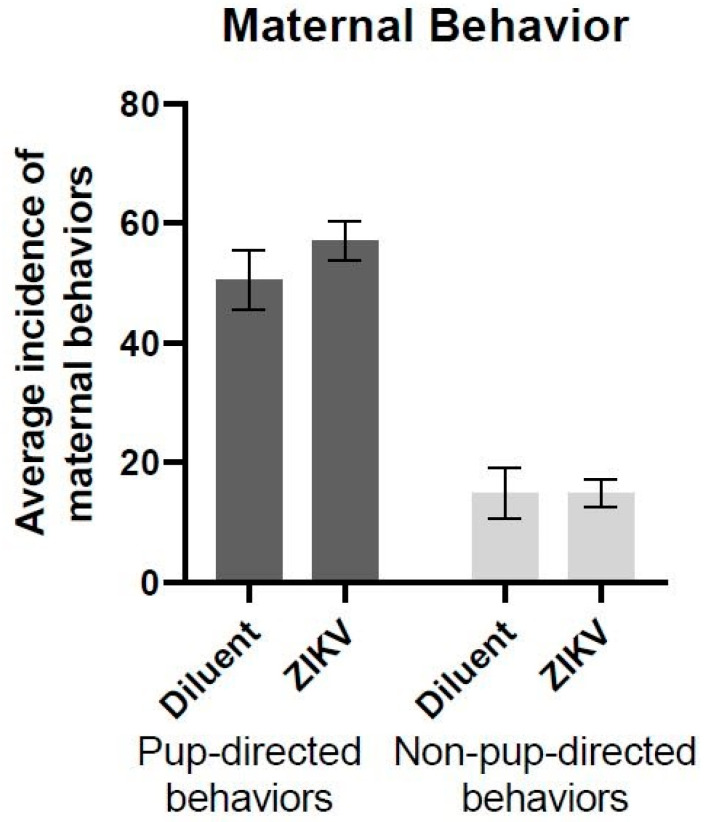
Average instance of pup-directed and non-pup-directed maternal behavior. There were no significant differences in pup-directed or non-pup-directed maternal behavior between infection groups (pup-directed: *F*(1,16) = 1.178, *p* = 0.294; non-pup-directed: *F*(1,16) = 3.57, *p* = 1.00) (*n* = 9 in each group).

We assessed whether maternal ZIKV infection would alter maternal behavior 6 days post-inoculation. For each animal, the sum of the 30 min morning and afternoon observation sessions (one hour total) was calculated and averaged across groups. A one-way ANOVA with infection as a factor (ZIKV *n* = 9 vs. diluent *n* = 9) revealed no significant effect of infection for either pup-directed behaviors (*F*(1,16) = 1.178, *p* = 0.294) or non-pup-directed behaviors (*F*(1,16) = 0.00, *p* = 1.00) (Figure 1).

### 3.2. Rotarod

**Figure 2:**
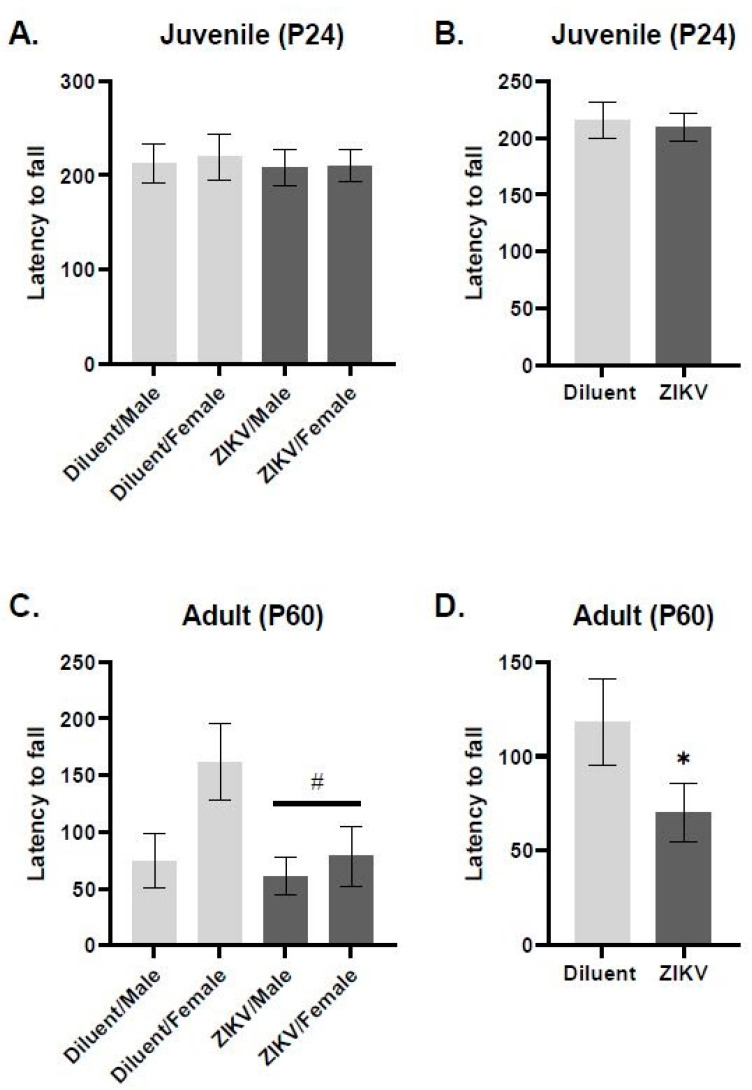
(**A**,**C**) groups separated by sex; (**B**,**D**) groups collapsed across sex. There is no significant effect of infection or sex in rotarod task in the juvenile offspring (**A**,**B**). At the adult time point (P60), there is a significant main effect of infection, where the rats prenatally exposed to ZIKV had a significantly shorter latency to fall compared to diluent control rats (*F*(1,30) = 4.502, *p* = 0.019); (**D**). When sex is included as a variable, there is a trend toward an infection x sex interaction (*F*(1,29) = 1.767, *p* = 0.057) (separated by sex: *n* = 8–10 animals in each group, collapsed across sex: *n* = 16–20 per group). * Denotes *p* < 0.05 relative to controls, # denotes *p* < 0.07 relative to diluent controls.

The ROT task was used to assess motor coordination in ZIKV and control rats at the juvenile and adult age. We found that at the juvenile time point (P24), there was no effect or interaction of prenatal ZIKV infection nor sex influenced performance on the ROT task (Figure 2A,B). However, at the adult time point (P60), there is a significant main effect of infection, where the rats prenatally exposed to ZIKV had a significantly shorter latency to fall compared to diluent control rats (*F*(1,30) = 4.502, *p* = 0.019, Figure 2D). When sex is included as a variable, there is a trend toward an infection x sex interaction (*F*(1,29) = 1.767, *p* = 0.057, Figure 2C). This interaction seems to be driven by the female rats, who perform the task well, but who exhibit significant deficits following prenatal ZIKV infection. Specifically, post hoc analysis revealed that females prenatally exposed to ZIKV have a significantly lower latency to fall compared to control females (*p* = 0.012). Regardless of prenatal infection, males do not differ in their performance on the ROT task (*p* = 0.715).

### 3.3. Immunohistochemistry

**Figure 3:**
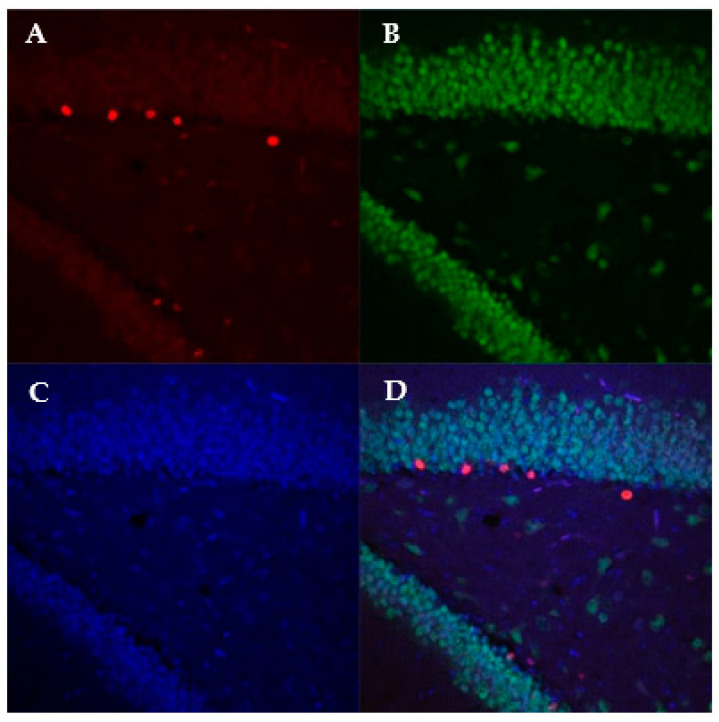
Representative of image of BrdU (**A**), NeuN (**B**), DAPI (**C**), and combined (**D**) fluorescent stain in the dentate gyrus at 20× magnification.

**Figure 4:**
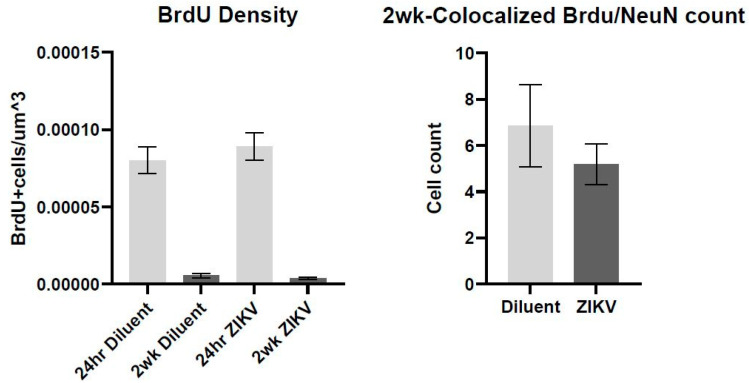
There was a main effect of time in BrdU density between the 24 h and two-week time point following BrdU injection (*F*(3,23) = 47.26; *p* < 0.001) where the two-week time point had fewer BrdU+ cells. There was no significant difference in BrdU and NeuN colocalized cells at 2 weeks (*F*(1,11) = 4.710; *p* = 0.446). *n* = 5–7 per group.

Using immunofluorescence and the software Imaris, we calculated the density of BrdU-labeled cells at 24 h and 2 weeks, as well as analyzed the density of BrdU and NeuN colocalized cells at 2 weeks (representative image, Figure 3). At the 24 h time point, we did not see a main effect of ZIKV infection on BrdU density (*F*(1,12) = 0.155; *p* = 0.523, Figure 4), suggesting that prenatal ZIKV infection on E18 does not result in alterations in cellular proliferation in the adolescent offspring. Further, survival of these new cells is not affected by gestational ZIKV infection, as there was no main effect of infection on the number of BrdU+ cells measured in the brain at two weeks (infection: *F*(1,11) = 3.314; *p* = 0.430, Figure 4). There was a main effect of time point on BrdU+ density where the two-week time point had significantly fewer cells than the 24 h time point regardless of prenatal infection (time point: *F*(3,23) = 47.26; *p* < 0.001). There was no difference in BrdU and NeuN colocalized cells at 2 weeks (*F*(1,11) = 4.710; *p* = 0.446), suggesting that E18 ZIKV infection did not affect the survival of new neurons.

### 3.4. RT-qPCR

#### 3.4.1. ZIKV Presence and Persistence

Here, we used qRT-PCR to measure ZIKV in the brain and serum of offspring that were prenatally exposed to ZIKV at three different time points, P2, P24, and P60. We used a standard curve generated by a 10-fold serial dilution of synthetic ZIKV RNA (ATCC) to determine viral presence and load in these tissues. At all three time points analyzed, we did not see evidence of viral presence in the serum or brain of offspring. We included positive controls made up of serum spiked with ZIKV RNA, as well as serum from P1 rat pups directly injected with 107 ZIKV and collected 24 h, 48 h, and 4 days after the injection. These positive controls positively showed up on the standard curve, validating the method. These results suggest that ZIKV RNA is not present in the brain or serum of offspring at P2, P24, or P60.

#### 3.4.2. Analysis of Gene Expression following Treatment with Poly(I:C)

**Figure 5:**
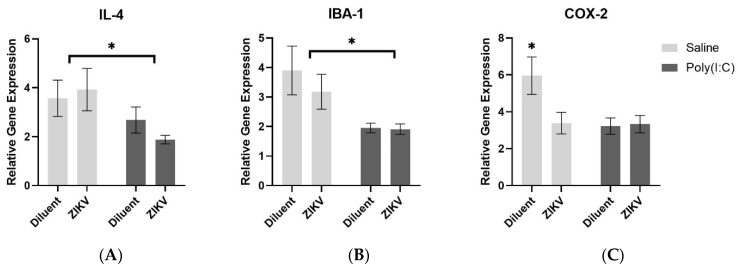
Hippocampus gene expression. There was a main effect of poly(I:C) for *IL-4* (**A**) and *Iba1* (**B**), where poly(I:C) exposure resulted in significantly lower gene expression compared to saline controls (*IL-4*: *F*(1,37) = 5.422, *p* = 0.025; *Iba1*: *F*(1,35) = 9.596, *p* = 0.004). There was an infection x poly(I:C) interaction for *COX-2* gene expression (**C**) where rats that received prenatal ZIKV infection, poly(I:C) injection, or both, had significantly lower levels of *COX-2* compared to controls (*COX-2*: *F*(1,38) = 18.695, *p* = 0.048); *n* = 10–12 per group. * denotes *p* < 0.05.

We measured the expression of immune molecules in the brain and the periphery at 6 h following treatment with poly(I:C), which stimulates the Toll-like receptor 3. We measured the expression of *Il-1β*, *IL-6*, *IL-4*, *TNF-α*, *IFN-β*, *Iba1*, and *COX-2* in the spleen and hippocampus at P60 (adulthood). In the hippocampus, there was a main effect of poly(I:C) for *IL-4* and *Iba1*, where poly(I:C) exposure resulted in significantly lower gene expression compared to controls (*IL-4*: *F*(1,37) = 5.422, *p* = 0.025; *Iba1*: *F*(1,35) = 9.596, *p* = 0.004, Figure 5A,B). There was an infection x poly(I:C) interaction for *COX-2* gene expression where rats that received prenatal ZIKV infection, poly(I:C) injection, or both, had significantly lower levels of *COX-2* compared to controls (*COX-2*: *F*(1,38) = 18.695, *p* = 0.048, Figure 5C).

**Figure 6:**
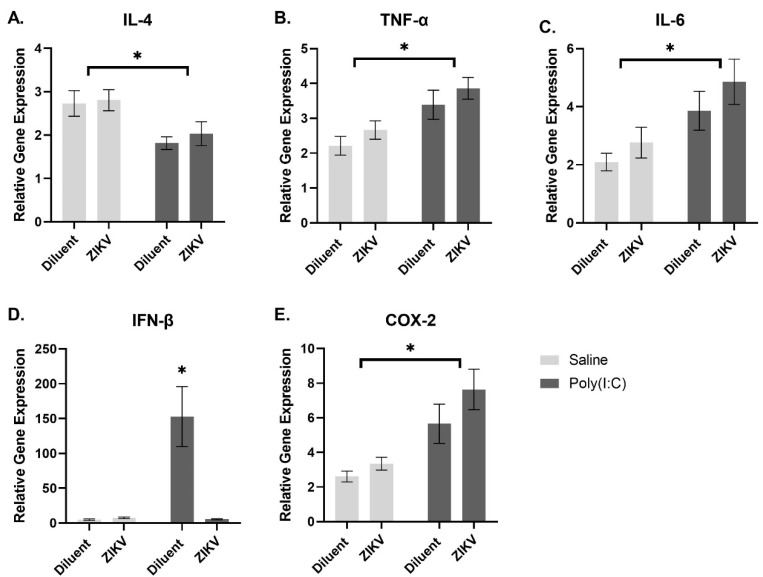
Spleen gene expression. poly(I:C) exposure resulted in increased gene expression of *TNF-α* (**B**), *IL-6* (**C**), and *COX-2* (**E**), and a reduced gene expression of *IL-4* (**A**) compared to controls (*TNF-α*: *F*(1,43) = 15.559, *p* < 0.001; *IL-6*: F(1,40) = 12.333, *p* = 0.001; *COX-2*: *F*(1,42) = 20.032, *p* < 0.001; *IL-4*: *F*(1,42) = 7.049, *p* = 0.011). In the spleen, there was an infection x poly(I:C) interaction whereby diluent rats that received poly(I:C) demonstrated a robust increase in *IFN-β* (**D**) gene expression; however, rats that were prenatally exposed to ZIKV, and then received poly(I:C), produced levels of *IFN-β* similar to the rats that did not receive poly(I:C) (*IFN-β*: *F*(1,39) = 8.067, *p* = 0.007), *n* = 10–12 per group. * Denotes *p* < 0.05.

In the spleen, poly(I:C) exposure resulted in increased gene expression of *TNF-α*, *IL-6*, and *COX-2*, and a reduced gene expression of *IL-4* compared to controls (*TNF-α*: *F*(1,43) = 15.559, *p* < 0.001; *IL-6*: *F*(1,40) = 12.333, *p* = 0.001; *COX-2*: *F*(1,42) = 20.032, *p* < 0.001; *IL-4*: *F*(1,42) = 7.049, *p* = 0.011, Figure 6A–C,E). There was an infection x poly(I:C) interaction whereby diluent rats that received poly(I:C) demonstrated a robust increase in *IFN-β* gene expression; however, rats that were prenatally exposed to ZIKV, and then received poly(I:C), produced levels of *IFN-β* similar to the rats that did not receive poly(I:C) (*IFN-β*: *F*(1,39) = 8.067, *p* = 0.007, Figure 6D).

## 4. Discussion

If a woman becomes infected with ZIKV during her pregnancy, her immune system may not be able to prevent the teratogenic virus from reaching her fetus, resulting in devastating changes to her child’s development [45]. Microcephaly is the most striking, and widely studied, outcome associated with prenatal ZIKV infection; however, only a fraction of babies born to mothers infected with ZIKV are microcephalic [46]. Babies born without microcephaly are not necessarily “out of the woods”, as more ‘subtle’ consequences of ZIKV have emerged including increased risk of seizures, irritability, and cognitive developmental delays [11,12]. The population of children prenatally exposed during the 2015 Brazilian outbreak, would now be reaching the age of 5 or 6. Thus, the full spectrum of long-term biological and behavioral consequences associated with congenital ZIKV exposure have not been established.

Various pieces of evidence support the notion that prenatal infection could lead to long-term deficits in the brain and behavior, for instance it has been shown that prenatal infection leads to a higher risk for developing neurological disorders including schizophrenia, autism, bipolar disorder, and cerebral palsy later in life [47] A primary mechanism by which ZIKV alters brain development is by preferentially infecting neural progenitor cells [2,6,8,9]. Disruption of the NPC population during development has been shown to lead to severe impairments in cognition including spatial recognition, learning and memory, emotional regulation, as well as later onset mental health disorders such as schizophrenia and depression [31]. Another way in which ZIKV could impact the fetus is by interfering with the developing immune system. While severe infection can result in direct neurological injury, mild-to-moderate infection can lead to less obvious consequences, such as altering the individual’s vulnerability to a subsequent immune challenge [48]. Given this evidence, we have reason to believe that prenatal ZIKV could lead to negative long-term consequences in the offspring. Therefore, using our rat model of prenatal ZIKV infection, we investigated the effect of congenital ZIKV infection on long-term neurological, immunological and behavioral outcomes.

Specifically, the ROT task uncovered a long-term motor deficit where adult rats infected with ZIKV prenatally fell off the apparatus significantly faster than controls. This appears to be mediated in a sex-specific manner, as females are primarily affected by prenatal infection, reducing their capacity to complete the task at the level of males. Although sex differences in behavior have not yet been reported in humans following congenital ZIKV infection, differences in behavior may emerge when the current population of children prenatally exposed reach adolescence [49]. Since there were no differences in maternal behavior, we can conclude that the differences we see between ZIKV and diluent groups are not due to impairments in maternal care. The direct mechanism by which ZIKV affects motor ability has been largely understudied; however, Cui and colleagues, who also found a ZIKV-induced rotarod deficit, showed that ZIKV infection led to a decrease in cerebellum size as well as a reduction in Purkinje cell number in ZIKV infected mice [50]. Neuroimaging studies in humans have also reported hypoplasia of the cerebellum in ZIKV infected infants [51]. As the cerebellum is integral for the coordination of movement, it is very possible that disruption of its development would account for the motor deficits we see in adulthood.

Our results suggest that ZIKV exposure on E18 does not affect cell proliferation, neuronal differentiation, or survival of new cells in the dentate gyrus of the hippocampus. We found decreased BrdU+ density at the two-week time point compared to the 24 h time point in both diluent and ZIKV offspring. Reduced BrdU+ density between the 24 h and two-week time point is consistent with other literature, as BrdU+ cells naturally die over time [52]. It is possible that with one injection on each day, the newly proliferated cells were not fully saturated, obscuring any differences in proliferation. Evidence from an in vitro model of ZIKV infection demonstrates an impairment in NPC synaptogenesis and an aberrant pattern of neurogenesis following infection with ZIKV [53]. As we did not find evidence of viral RNA in the serum or brain of offspring, it is possible the timing of infection, strain, or virus titer was not optimal to induce neural infection of offspring, and thereby alter neurogenesis. Therefore, the effects we see in motor coordination may represent indirect consequences of maternal immune activation [54].

It has been also demonstrated that maternal infection can interfere with typical development of the immune system and the brain [55]. While severe infection can result in direct neurological injury, mild-to-moderate infection can lead to less obvious consequences, such as altering the individual’s vulnerability to a subsequent immune challenge [48]. The “two-hit hypothesis of immune activation” posits that immune activation during a sensitive period of development primes the later-life immune system to mount an exaggerated response following a second immune challenge [23]. This exaggerated response includes the overproduction of cytokines in the brain, which is known to lead to various cognitive deficits, including those of memory, cognition, and the susceptibility to mental health disorders such as anxiety and depression [23,36]. The effect of prenatal ZIKV infection on the developing immune system has not yet been explored. Here, we were interested in understanding whether prenatal ZIKV permanently altered the offspring’s immune response to a secondary viral immune challenge in adulthood.

Largely, prenatal administration of ZIKV did not affect the immune system’s ability to respond to poly(I:C) via the expression of most immune molecules analyzed. However, there was a robust effect in *IFN-β* gene expression between prenatally exposed rats and controls, where prenatal exposure to ZIKV prevented an *IFN-β* response to a viral challenge in adulthood. To our knowledge, this is the only study of its kind to assess the immune response later in life following prenatal ZIKV infection. This result suggests that ZIKV has a long-lasting effect on the offspring’s immune system, specifically to their IFN response. As the IFN system is the first line of defense for a response against viral infection, lacking this response could leave these individuals more susceptible to viral infection. More research will need to be completed to fully elucidate the underlying mechanisms responsible for the reduction in *IFN-β*.

Here, we used our rat model of prenatal ZIKV infection to investigate the long-term impact on the health of offspring. We found that adult offspring that were prenatally infected with ZIKV exhibited motor deficits in a sex-specific manner and failed to mount a normal interferon response to an immune challenge later in life.

The public health community was not prepared for the 2015 Brazilian outbreak, and could not have predicted the dramatic developmental consequences that it caused. However, the scientific response was immense, resulting in the WHO to declare a public health emergency of international concern and over 6000 scientific publications to be published. Even so, we still lack a complete understanding of ZIKV’s ability to cause disease, as well as a therapeutic solution. The largest gap in knowledge, and perhaps the most pressing, is our understanding of the long-term impacts that ZIKV has on the brain and the immune system. There is likely an unrecognized population of children that could be susceptible to long-term effects of congenital exposure. While the Brazilian epidemic of 2015 has since subsided, the potential threat of another ZIKV outbreak is on the horizon. Due to climate change and increased urbanization, the *Aedes aegypti* mosquito population is predicted to spread to cover most of the world by 2050 [5]. In order to help individuals already exposed to ZIKV, as well as be prepared for future outbreaks, we need to understand the full spectrum of neurological and immunological consequences that could arise following prenatal exposure.

## Figures and Tables

**Table 1:** Rat primers used for quantitative real-time PCR.

Gene	Forward (F) and Reverse (R) Primers
*IFN-* *β*	F: ATGGCCAACACGTGGACCCT R: TCAGTTCTGGAAGTTTCTAT
*IL-4*	F: AAGGAACACCACGGAGAACG R: CAGACCGCTGACACCTCTAC
*Iba1*	F: GAATGATGCTGGGCAAGAGA R: CAGTTGGCTTCTGGTGTTC
*COX-2*	F: CTTCGCCTCTTTCAATGTGC R: GGTCAGTAGACTCTTACAGC
*TNF-* *α*	F: CTTCAAGGGACAAGGCTG R: GAGGCTGACTTTCTCCTG
*ZIKV*	F: CCGCTGCCCAACACAAG R: CCACTAACGTTCTTTTGCAGACAT Probe: FAM/AGCCTACCT/ZEN/TGACAAGCAATCAGACACTCAA/3IABkFQ

## Data Availability

Data can be made available upon request.
